# Dengue viral infection in Indonesia: Epidemiology, diagnostic challenges, and mutations from an observational cohort study

**DOI:** 10.1371/journal.pntd.0007785

**Published:** 2019-10-21

**Authors:** I Made Susila Utama, Nurhayati Lukman, Dewi Dian Sukmawati, Bachti Alisjahbana, Anggraini Alam, Dewi Murniati, I Made Gede Dwi Lingga Utama, Dwiyanti Puspitasari, Herman Kosasih, Ida Laksono, Muhammad Karyana, Mulya Rahma Karyanti, M. M. D. E. A. H. Hapsari, Ninny Meutia, C Jason Liang, Wahyu Nawang Wulan, Chuen-Yen Lau, Ketut Tuti Merati Parwati

**Affiliations:** 1 Sanglah Hospital, Bali, Denpasar, Indonesia; 2 Indonesia Research Partnership on Infectious Disease (INA-RESPOND), Jakarta, Indonesia; 3 Faculty of Medicine, Universitas Padjadjaran, Hasan Sadikin Hospital, Bandung, West Java, Indonesia; 4 Sulianti Saroso Infectious Disease Hospital, Jakarta, Indonesia; 5 Department of Child Health, Faculty of Medicine, Dr. Soetomo Hospital, Airlangga University, Surabaya, Indonesia; 6 Sardjito Hospital, Yogyakarta, Indonesia; 7 National Institute of Health Research and Development (NIHRD), Ministry of Health Republic of Indonesia, Jakarta, Indonesia; 8 Cipto Mangunkusumo Hospital, Jakarta, Indonesia; 9 Kariadi Hospital, Semarang, Indonesia; 10 Wahidin Sudirohusodo Hospital, Makassar, Indonesia; 11 National Institute of Allergy and Infectious Disease (NIAID), National Institutes of Health, Bethesda, Maryland, United States of America; Oregon Health and Science University, UNITED STATES

## Abstract

**Background:**

Dengue virus (DENV) infection is a major cause of acute febrile illness in Indonesia. Diagnostic inaccuracy may occur due to its varied and non-specific presentation. Characterization of DENV epidemiology, clinical presentation, and virology will facilitate appropriate clinical management and public health policy.

**Methodology/Principal findings:**

A multicenter observational cohort study was conducted in Indonesia to assess causes of acute fever requiring hospitalization. Clinical information and specimens were collected at enrollment, 14–28 days, and 3 months from 1,486 children and adults. Total of 468 (31.9%) cases of DENV infection were confirmed by reference laboratory assays. Of these, 414 (88.5%) were accurately diagnosed and 54 had been misdiagnosed as another infection by sites. One hundred initially suspected dengue cases were finally classified as ‘non-dengue’; other pathogens were identified in 58 of those cases. Mortality of DENV infection was low (0.6%). Prior DENV exposure was found in 92.3% of subjects >12 years. DENV circulated year-round in all cities, with higher incidence from January to March. DENV-3 and DENV-1 were the predominant serotypes. This study identified DENV-1 with TS1_19_(C→T) substitution in the serotyping primer annealing site, leading to failure of serotype determination.

**Conclusions/Significance:**

DENV is a common etiology of acute febrile illness requiring hospitalization in Indonesia. Diagnostic accuracy at clinical sites merits optimization since misdiagnosis of DENV infection and over-estimation of dengue can negatively impact management and outcomes. Mutation at the annealing site of the serotyping primer may confound diagnosis. Clinicians should consider following diagnostic algorithms that include DENV confirmatory testing. Policy-makers should prioritize development of laboratory capacity for diagnosis of DENV.

## Author summary

Dengue is a mosquito-borne viral disease whose global incidence has increased dramatically in recent decades. Infection with any of the four serotypes can cause subclinical to life-threatening illness. Indonesia and other subtropical regions are hyper-endemic, thus are at increased risk of disease impact. We conducted a multicenter observational cohort study of acute febrile illness requiring hospitalization in children and adults in Indonesia from 2013–2016. Epidemiology, clinical course, and virology of dengue were assessed. Dengue infection is the main cause of hospitalized acute febrile illness in Indonesia. DENV serotypes 1–4 circulated throughout the study period, with highest overall rates in January to March. We observed DENV-1 with TS1_19_(C→T) substitution in the serotyping primer annealing site that caused failure of routine detection. Diagnostic accuracy at clinical sites merits optimization. Clinicians should consider following diagnostic algorithms that include DENV confirmatory testing, while policy-makers should prioritize development of laboratory capacity for diagnosis of DENV.

## Introduction

Dengue Virus (DENV) infection is a global health threat that can strain local economies and healthcare resources. Previously unaffected countries are increasingly reporting outbreaks. Only a few countries in Europe and Antarctica have thus far evaded vector borne transmission of DENV [1]. Actual rates of DENV infection are likely under-reported and many cases misclassified [1]. It was recently estimated that 390 million (95% CI: 284–528 million) DENV infections occur annually and 96 million (95% CI: 67–136 million) were symptomatic [2]. Models suggest that by the year 2085, half of the world population may be living in areas at risk of dengue transmission [3]. Indonesia is a DENV endemic region and has experienced a 700-fold increase in incidence over the past 45 years [4]. A clear understanding of the current epidemiology of DENV in Indonesia is critical for design of appropriate public health measures.

DENV infection has a wide range of clinical presentations, from subclinical to debilitating but transient Dengue Fever (DF) to potentially life-threatening Dengue Hemorrhagic Fever (DHF) and Dengue Shock Syndrome (DSS). Atypical presentations are categorized as expanded dengue syndrome [5]. Diagnosis of DENV infection in Indonesia is typically based on clinical presentation, common laboratory evaluation, and rapid diagnostic tests. Specific DENV laboratory confirmation is not usually pursued. Since care for DENV infection is supportive, diagnostic inaccuracy can result in inappropriate treatment, including administration of unnecessary antibiotics for cases attributed to other infections and forgoing of needed antibiotics when other infections are presumed to be dengue. Inappropriate clinical management and inappropriate use of antimicrobials may contribute to increased morbidity, mortality and treatment cost, as well as promote antibiotic resistance.

To better understand the current epidemiology of dengue in Indonesia, cases of presumed and laboratory identified dengue from a cohort study on febrile illnesses requiring hospitalization in Indonesia were characterized. Approaches for assessing dengue infection, genetic characterization, and their public health implications were considered.

## Methods

### Participants

Patients presumed to have DENV infection based on clinical presentation or found to have DENV infection by subsequent laboratory testing were identified from the Etiology of Acute Febrile Illness Requiring Hospitalization (AFIRE) cohort study, conducted by the INA-RESPOND (Indonesia Research Partnership on Infectious Diseases) network [6] in Indonesia from 2013 to 2016. The AFIRE study recruited patients who presented to hospitals for evaluation of acute fever, were at least one year old, were hospitalized within the past 24 hours, and had not been hospitalized within the past three months. Clinical information and biological specimens were collected at enrollment, 14–28 days after enrollment, and three months after enrollment.

### Sites

**Fig 1:**
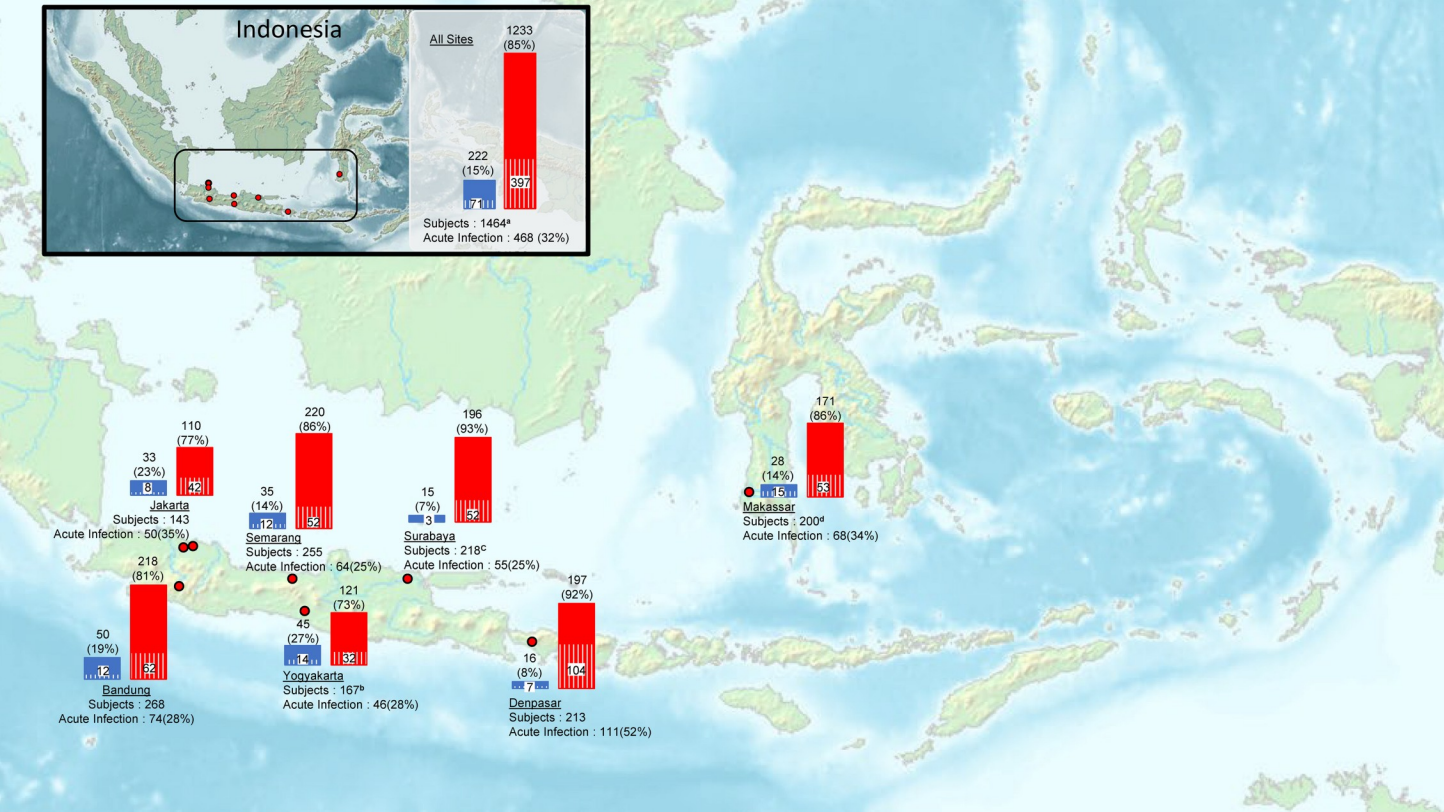
Geographic distribution and rates of DENV cases. Red dots show study site location. The number of fever cases and acute Dengue infections (percent) at each site are shown below the site name. Bars show the proportion of patients with (red bars) and without (blue bars) prior exposure; white stripes pattern inside the bars shows the subset who experienced acute infection. Note: Nine subjects^a^ had no acute specimens for exposure prior to enrollment: 1 subject in Yogyakarta^b^, 7 subjects in Surabaya^c^, and 1 subject in Makassar^d^. Map source: Wikimedia Commons Atlas of the World *[Atlas of Indonesia]*. Available from: https://commons.wikimedia.org/wiki/Atlas_of_Indonesia#/media/File:Map_of_Indonesia_Demis.png [Accessed 23 September 2019].

Patients were recruited to the AFIRE study from eight tertiary hospitals in seven cities in Indonesia: Bandung, Denpasar, Jakarta, Makassar, Semarang, Surabaya and Yogyakarta (Fig 1). All study sites contributed cases by diagnosing DENV clinically and/or using laboratory diagnostics (NS1 and/or serology test and/or rapid diagnostic test using different manufacturer).

### Testing algorithm for dengue diagnosis at INA-RESPOND reference laboratory

Subjects with acute and convalescent (at least two-weeks apart) plasma were first screened for dengue infections using DENV IgM and IgG by ELISA, followed by DENV NS1 and RT-PCR. Acute plasma from subjects with single specimens were tested using DENV IgM and IgG by ELISA, DENV NS1 and RT-PCR.

### Reference laboratory procedures

#### Enzyme-linked Immunosorbent Assay (ELISA)

Assays were conducted using Focus Dengue IgM capture/IgG indirect and Antigen NS1 ELISA (Focus Diagnostics, CA, USA). This is a qualitative assay targeting IgM and IgG antibodies to DENV and NS1 DENV antigen. Absorbance was measured at a wavelength of 450 nm on a microplate reader. The index value was calculated by dividing the sample absorbance by OD cut-off. Index <1.0 was considered negative, Index >0.9 to <1.0 was considered borderline, and Index >1.0 was considered positive [7].

#### Viral RNA Extraction

Viral RNA was extracted from 140 μl plasma using the QIAamp Viral RNA Mini Kit (Qiagen, Hilden, Germany). Viral RNA was eluted in 60 μl of AVE buffer.

#### Dengue serotype determination using the multiplex semi-nested reverse transcription polymerase chain reaction (Msn RT-PCR)

Dengue serotypes were determined by Msn RT-PCR [8] targeting a region encompassing the C-prM gene of DENV with the One-Step RT-PCR Kit (Qiagen, Hilden, Germany) in an Applied Biosystems ProFlex PCR System (Thermo Fisher Scientific, MA, USA). The Msn RT-PCR product was visualized in a 1.5% agarose gel electrophoresis alongside a 100-bp DNA ladder (Invitrogen, CA, USA). The amplicon size for determination of serotypes are 482 bp, 119 bp, 290 bp, and 392 bp for DENV-1, DENV-2, DENV-3, and DENV-4 [8], respectively.

#### Dengue genotype determination based on nucleotide sequencing of the Envelope (E) gene

Specimens positive by Msn RT-PCR were selected randomly based on serotype distribution at each site to represent geographical and temporal distribution for sequencing using methods that have been published previously [9–11]. Viral RNA was converted to complementary DNA (cDNA) using the SuperScript III First-Strand Synthesis System for RT-PCR (Invitrogen, CA, USA) according to the manufacturer’s instructions, then amplified by PCR at a region covering the structural (*capsid-pre-membrane/membrane-envelope* (C-prM/M-E)) genes using the Platinum^TM^ SuperFi^TM^ DNA Polymerase (Invitrogen, CA, USA). The amplification primers consist of D1F751 and D1R2581 for DENV-1 [9], D2F798 and D2R2516 for DENV-2 [10], D3F791 and D3R2492 for DENV-3 [9], and D4s1c and D4a18 for DENV-4 [12], which resulted in DNA fragments of sizes 1831-bp, 1719-bp, 1702-bp, and 2550-bp for DENV-1, DENV-2, DENV-3, and DENV-4, respectively. DNA sequencing was performed on the purified PCR product by a contracted company (1st Base, Malaysia) using sequencing primers covering the structural region of dengue viruses (S1 Table). Chromatograms were edited and assembled using BioEdit 7.2.5 software. Phylogenetic trees describing genotypes within each of DENV serotypes were built using the maximum likelihood (ML) method, based on the *E* gene of DENV, in the Molecular Evolutionary Genetics Analysis version 7 (MEGA 7.0.20) software. The genotype classification scheme was previously described for each serotype [11, 13–15]. Models of nucleotide substitution that best described sequence evolution for each serotype were identified as TN93+G+I for DENV-1 and DENV-2 data sets and TN93+G for DENV-3 and DENV-4 data sets. The strength of tree topology was estimated by bootstrap analysis using 1000 replicates. Trees were unrooted.

#### Characterization of DENV mutations

For serotypes that could not be determined with the standard primer set, the TS1_19_(C→T) substitution at primer annealing site was identified. Then a modified TS1: 5’-CGT CTC AGT GAT CCG GGG RC-3’ [16] was employed to overcome failure of Msn RT-PCR detection.

### Assessment of Dengue infection and clinical categories

Recent dengue virus infection was confirmed when viral RNA and/or NS1 antigen was detected in an enrollment sample and/or IgM and IgG seroconversion or increased optical density was observed between enrollment and a subsequent time point (14–28 days apart). Positive DENV IgG in acute specimens was considered evidence of DENV exposure prior to enrollment. Confirmed DENV infections were considered primary when DENV IgG antibodies were not detected in acute specimens; and infections were considered secondary when IgG was detected in acute specimens.

Dengue cases were classified according to the 2011 WHO guidelines [5]. Based on plasma leakage (hemoconcentration ≥20%), thrombocytopenia <100,000/mm^3^, and hemorrhagic manifestations, cases were categorized as DF, DHF I, DHF II, DHF III, or DHF IV. Unusual manifestations such as neurological, hepatic, renal, and other isolated organ involvement were also noted. For analysis purposes, cases were grouped into DF; DHF I and II; or DHF III, IV, and atypical manifestations.

### Analysis

All DENV cases confirmed by the reference laboratory were stratified by serotype, and further stratified by year and location; the absolute number of cases in each stratum was reported. Cases that were confirmed by the reference laboratory but missed at the study sites were stratified by the final study site diagnosis; within each stratum, clinical characteristics were summarized using absolute counts and percentages. The difference in rates of severe cases between secondary and primary infections was summarized with a point estimate, and the p-value was calculated using a chi-squared test. Discordant diagnoses were defined as those who were diagnosed with dengue at a site but did not have reference laboratory confirmed dengue, or those who did have reference laboratory confirmed dengue but were not diagnosed with dengue at a site. The contrast in rates of clinically DF patients between missed cases (those with reference laboratory confirmed dengue who were not initially diagnosed with dengue at a site) and correctly diagnosed cases (those with reference laboratory confirmed dengue who were correctly diagnosed with dengue at a site) was summarized with an odds ratio and 95% confidence interval from Fisher’s exact test. Statistical analyses were performed using Stata 15.1 (StataCorp LLC).

### Ethics

The AFIRE study received ethical approval from the IRBs of Faculty of Medicine University of Indonesia/ Cipto Mangunkusumo Hospital (451/PT02.FK/ETIK/2012), Dr. Soetomo Hospital (192/Panke.KKE/VIII/2012), and the National Institute of Health and Research and Development (NIHRD), Ministry of Health, Indonesia (KE.01.05/EC/407/2012). All eligible participants or their legal guardian signed consent before enrolled to the study.

## Results

### Epidemiology

Four hundred sixty-eight of 1,464 subjects (32%) enrolled in the AFIRE study from 2013 to 2016 had confirmed DENV infection as the cause of acute fever (Fig 1). Three hundred sixty-three cases had positive serology and PCR/NS1; 70 cases with only acute specimens available were positive by PCR/NS1; and 35 cases had positive serology only. From 468 cases, 364 cases were identified from 1,158 subjects who had paired specimens, and 104 cases from 306 subjects who only had single specimens. Of 202 subjects who had no evidence of dengue infections, other pathogens were identified in 75 subjects.

**Fig 2:**
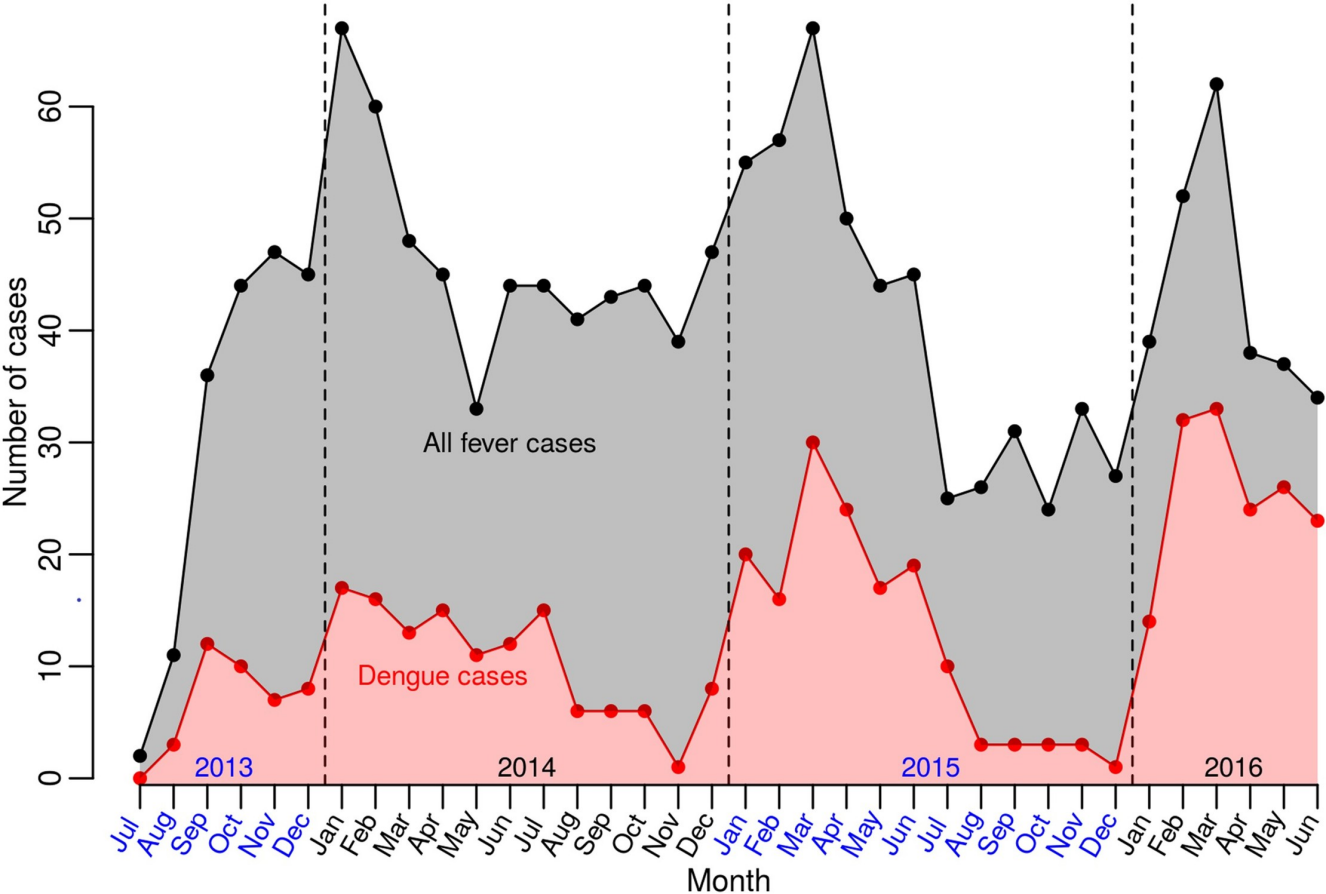
Dengue cases per month. Red dots/area: dengue cases. Black dots/gray area: all fever cases.

Amongst the seven study cities, the highest proportion of DENV cases was diagnosed at Denpasar (52%) and followed by Makassar (34%). Other cities had lower rates of DENV (Fig 1). DENV cases occurred year-round, but were more common from January to March (Fig 2). The high proportion of dengue infection was consistent across study years, age groups, genders, and sites.

DENV patients tended to be young (mean age 19 ±12.4 years) and were more likely to be male (non-significant trend), generally reflecting the AFIRE study population. Clinical syndrome did not differ by dengue serotype (S2 Table).

**Fig 3:**
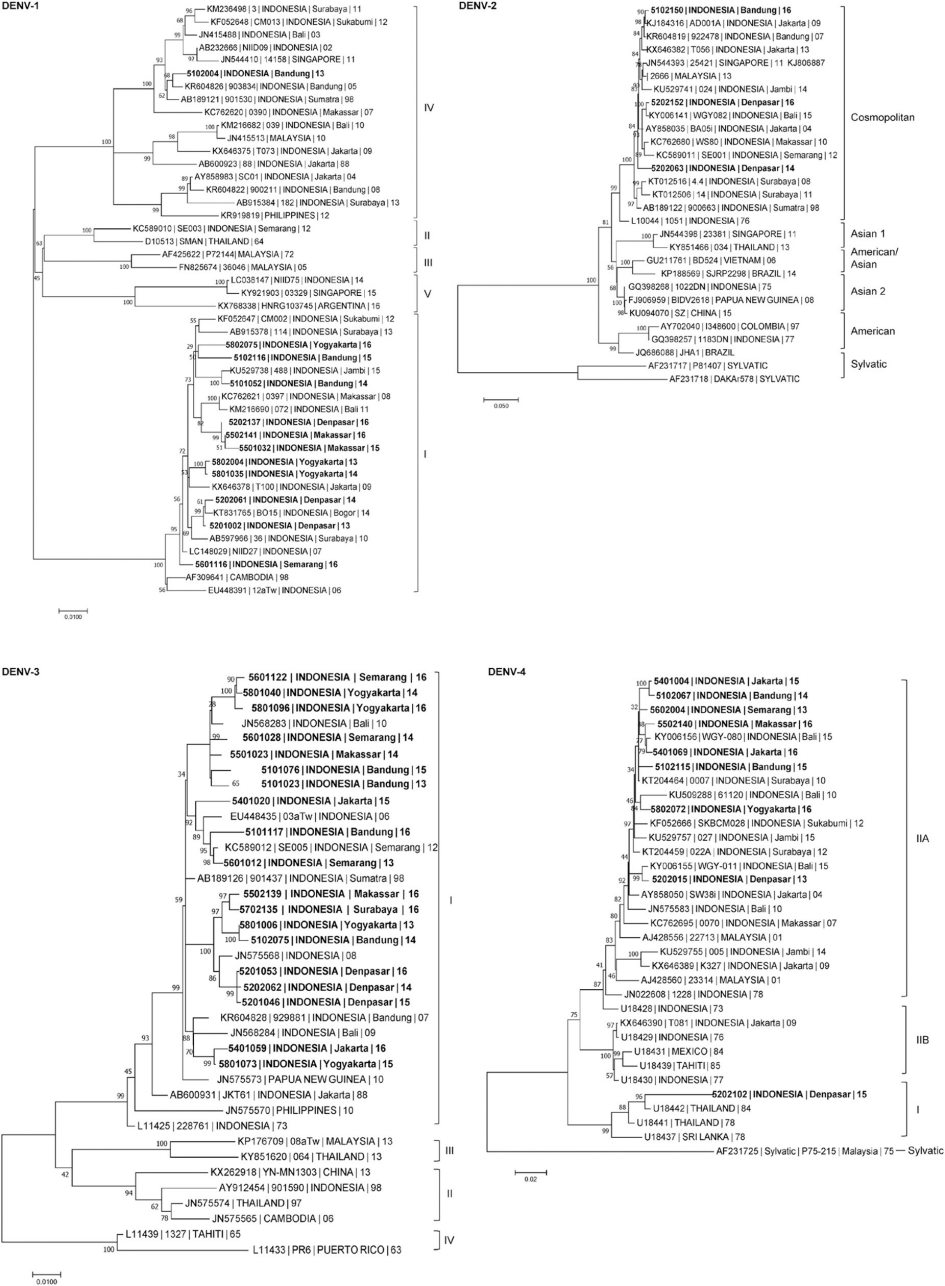
Phylogenetic trees showing the relationship of DENV identified in AFIRE study. The AFIRE DENV *envelope* (E) gene sequences are presented as: Subject Identification Number | Country | City | Year (in bold). The reference E gene sequences retrieved from GenBank are presented as: Accession Number | Strain (if available) | Country | City (if available) | Year (not bold). The scale presents the number of nucleotide substitutions per site along the branches. The relationships were constructed by the maximum likelihood (ML) method using nucleotide sequences of the E gene (DENV-1, 2, 4 = 1485 bp; DENV-3 = 1479 bp), with 1000 bootstrap replicates. The trees consist of 43 E gene nucleotide sequences from AFIRE specimens (DENV-1 = 12, DENV-2 = 3, DENV-3 = 19, DENV-4 = 9) and other sequences from the GenBank database. The sequences of the AFIRE DENV E gene were submitted to GenBank under accession numbers MK629460 to MK629502. The clustering of genotypes for DENV-1, DENV-2, DENV-3, and DENV-4 referred to the classification that were described earlier [11, 13–15].

Serotypes 1–4 were found circulating during the entire study period with predominance of DENV-3 nationally and in most cities, except DENV-1 in Denpasar and DENV-2 in Surabaya. Monthly DENV serotype distributions by city show dynamic variation of predominant serotype (S1 Fig). Two patients were infected with two different serotypes (Table 1). Phylogenetic analysis of DENV-1 showed clustering in genotype I, but a specimen collected from Bandung in 2013 was genotype IV. DENV-2 specimens tended to cluster in genotype Cosmopolitan. DENV-3 specimens clustered in genotype I. DENV-4 specimens were mostly in genotype II, with a 2015 specimen from Denpasar falling within genotype I (Fig 3).

**Table 1:** Circulating DENV serotypes by study year and site.

	DENV-1 (n = 103)	DENV-2 (n = 84)	DENV-3 (n = 186)	DENV-4 (n = 21)	Dual serotype (n = 2)	Unknown serotype (n = 72)
**Year**						
2013	17	3	14	3		4
2014	34	23	37	3	DENV-1&2 (1)	28
2015	31	25	71	4	DENV-2&3 (1)	18
2016	21	33	64	11		22
**Location**						
Bandung	12	9	36	3	DENV-2&3 (1)	13
Denpasar	44	15	37	5		10
Jakarta	11	7	22	3		7
Makassar	9	10	33	4		12
Semarang	11	11	24	1		17
Surabaya	2	23	18	1	DENV-1&2 (1)	10
Yogyakarta	14	9	16	4		3

Two patients that had two different serotypes are not included in the individual DENV serotype columns.

### Clinical severity and mortality

Based on clinical signs and symptoms from 468 cases of DENV infection, 187 were classified as DF (40%); 270 as DHF grade I and II (57.7%); and 11 cases as DHF grade III and IV or atypical manifestations (2.3%). Seventy-one cases were primary and 397 were secondary infection. The severe cases were more common in secondary than primary infection. While the difference was not statistically significant, our findings add to the body of evidence that secondary infection cases are more prone to becoming severe (2.8% vs. 0%, p<0.15). Severe cases tended to be DENV-1 (3 cases) and DENV-3 (8 cases). Mortality due to DENV infection was low (3/468; 0.6%). The first case was a 2 year-old boy, with no underlying disease, who had a secondary dengue infection with DENV-3. He presented to the hospital with 6 days of fever, vomiting, ecchymosis, hemoconcentration (44%), leukopenia (3,800/mm^3^), and thrombocytopenia (34,000/mm^3^). He died on day 3 hospitalization with DSS. The second subject was a 23 year-old male with leukemia, presenting with fever, epistaxis, gum bleeding, nausea, leukocytosis (13,000/mm^3^), and thrombocytopenia (23,000/mm^3^). Clinically he was classified as DF with hemorrhagic manifestations. He was hospitalized for 8 days and died with septic shock. DENV-4 was retrospectively detected by the reference laboratory. The third case was a 62 year-old man with COPD who presented with pneumonia. He had normal leukocyte and platelet counts at enrollment. He died on hospital day 3 from complications of intra-cranial hemorrhage and septic shock. DENV-1 was detected in his blood by the reference laboratory.

### DENV seroprevalence

Almost half (78/194, 40.2%) of AFIRE study participants under age 5 years showed evidence of dengue exposure based on serologic testing. The proportion of patients with prior dengue exposure increased with age to approximately 90% in 12–25 year-old and almost 100% in adults over 25 years.

### Discordant diagnoses

**Fig 4:**
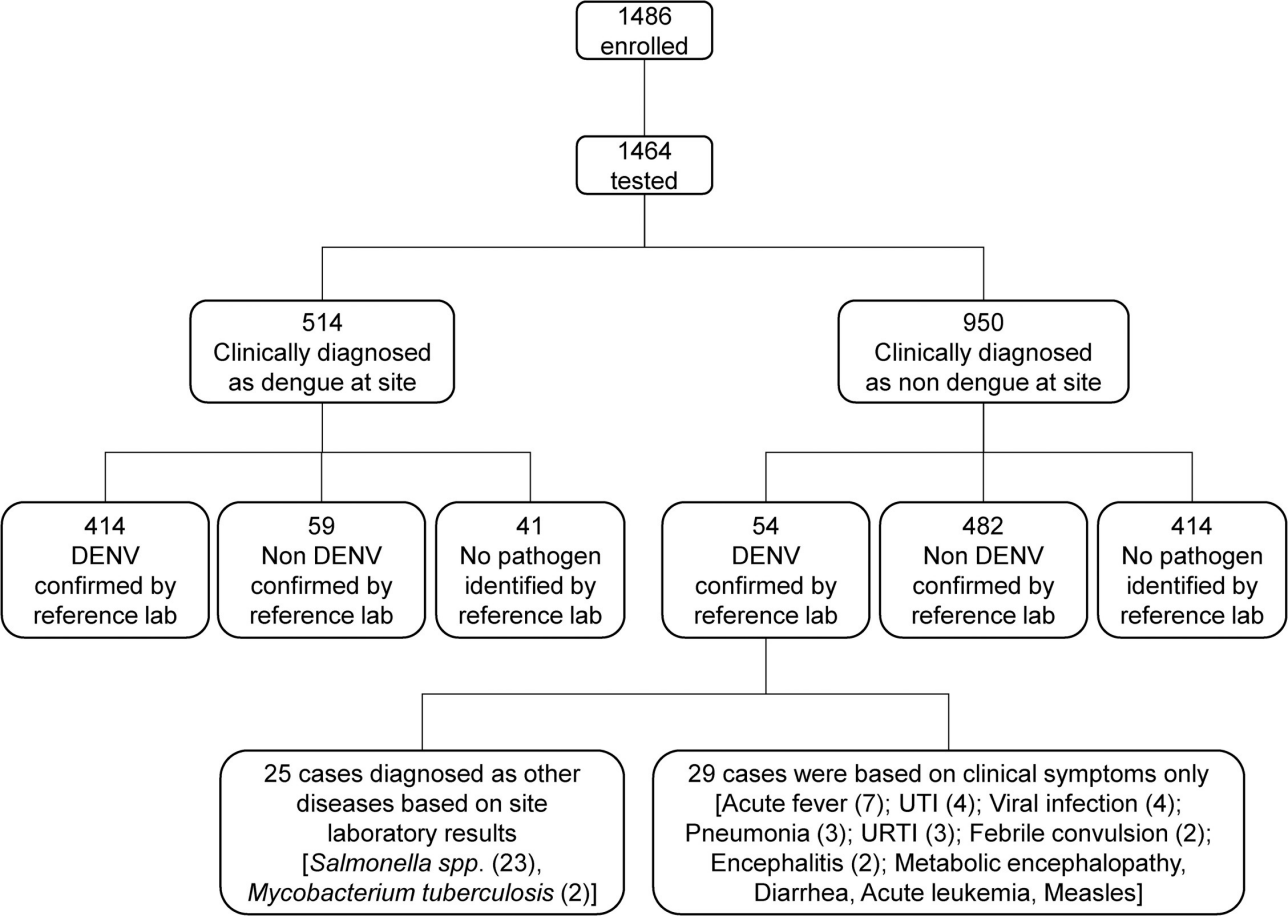
Clinical diagnoses and reference lab confirmation of DENV infections. Presumed etiologies of missed DENV infections and actual etiologies of incorrectly presumed DENV infections are shown. Reference laboratory diagnosis was considered the true diagnosis.

A total of 88.5% (414/468) of confirmed dengue cases was correctly diagnosed by study sites. In the 54 cases where dengue was misdiagnosed as other diseases, 25 cases (46%) were diagnosed based on site laboratory results and 29 cases (54%) were diagnosed by only clinical symptoms (Fig 4). Details on how these 25 cases were diagnosed on sites, dengue tests on sites, how we excluded on-site diagnoses and how dengue was confirmed for these cases are listed in Fig 4. Two cases that were diagnosed pulmonary tuberculosis at sites while dengue was confirmed by RT-PCR/NS1 and serology suggested acute dengue infection in subjects with chronic tuberculosis.

Of these 54 cases, dengue rapid tests were performed in 29 cases at sites. In four cases, NS1 was positive but typhoid fever was the site diagnosis as the Tubex test for IgM *Salmonella typhi* was also positive. In total, dengue was considered in the differential diagnosis in 10 of the 54 missed cases. Comorbidities were observed in 12 cases, including pulmonary tuberculosis, COPD, leukemia, thalassemia, chronic otitis, septal defect, and choledochal cyst. Subjects who were incorrectly diagnosed as non-dengue infection had higher odds of being DF than subjects who were correctly diagnosed as dengue infections; 81.5% of missed cases were clinically DF, whereas only 34.5% of those correctly diagnosed as DENV infection were DF (OR 8.3, 95% CI: 4–19.1).

Of the 514 cases clinically diagnosed as DENV infection by sites, 100 (19.5%) were classified by the reference lab as non-dengue; 58 were found to have another pathogen and no pathogen was identified in 42 cases (Fig 4). Other confirmed pathogens were *Rickettsia typhi* (20), chikungunya virus (11), *Leptospira spp* (10), *Salmonella typhi/paratyphi* (7), influenza A/B virus (5), measles virus (2), *Klebsiella pneumoniae* (1), entero-pathogenic *Escherichia coli* (1), and Seoul virus (1). Demography, clinical manifestations, hematology, and biochemistry profiles grouped by identified pathogen are shown in Table 2.

**Table 2:** Infection characteristics: Clinical signs and symptoms, hematology and biochemistry profiles.

Clinical signs and symptoms	Dengue (414)	Pathogen identified by reference laboratory					
		*R*. *typhi* (20)	Chikungunya (11)	Leptospira (10)	*S*. typhi (7)	Influenza A/B (5)	Others (5)
**Demography, n (%)**							
Age ≥ 18 y.o.	196 (47)	15 (75)	5 (45)	8 (80)	2 (29)	5 (100)	4 (80)
Gender : Male	247 (60)	12 (60)	7 (64)	9 (90)	5 (71)	3 (60)	2 (40)
**Lab confirmation, n (%)**							
PCR/BC and serology	299 (72)	12 (60)	8 (73)	8 (80)	4 (57)	0 (0)	5 (100)
Serology only	115 (28)	8 (40)	3 (27)	2 (20)	3 (43)	5 (100)	0 (0)
**Signs and Symptoms, n (%)**							
Fever	414 (100)	20 (100)	11 (100)	10 (100)	7 (100)	5 (100)	5 (100)
Nausea	314 (76)	15 (75)	8 (73)	7 (70)	5 (71)	5 (100)	4 (80)
Headache	241 (58)	16 (80)	5 (45)	4 (40)	4 (57)	3 (60)	2 (40)
Vomiting	225 (54)	7 (35)	5 (45)	6 (60)	4 (57)	3 (60)	3 (60)
Lethargy	85 (21)	4 (20)	2 (18)	2 (20)	3 (43)	3 (60)	2 (40)
Arthralgia	128 (31)	6 (30)	5 (45)	2 (20)	3 (43)	4 (80)	2 (40)
Myalgia	94 (23)	7 (35)	4 (36)	1 (10)	3 (43)	1 (20)	0 (0)
Chills	56 (14)	4 (20)	1 (9)	4 (40)	0 (0)	2 (40)	0 (0)
Epigastric pain	101 (24)	6 (30)	2 (18)	0 (0)	3 (43)	1 (20)	2 (40)
Cough	63 (15)	3 (15)	5 (45)	2 (20)	3 (43)	5 (100)	4 (80)
Diarrhea	38 (9)	1 (5)	2 (18)	4 (40)	2 (29)	0 (0)	2 (40)
Skin rash	7 (2)	0 (0)	0 (0)	0 (0)	0 (0)	0 (0)	2 (40)
Constipation	19 (5)	4 (20)	1 (9)	2 (20)	0 (0)	0 (0)	0 (0)
Altered mental status	2 (0.5)	0 (0)	0 (0)	0 (0)	0 (0)	0 (0)	0 (0)
Dysuria	7 (2)	0 (0)	0 (0)	0 (0)	0 (0)	0 (0)	0 (0)
Icteric	1 (0.2)	0 (0)	0 (0)	1 (10)	0 (0)	0 (0)	0 (0)
Triad Rickettsia: fever, headache and rash	5 (1)	0 (0)	0 (0)	0 (0)	0 (0)	0 (0)	1 (20)
**Hematology findings: Median (range)**							
Hb (mg/dl)	14.1 (5.5–20.8)	14.35 (11.4–16.2)	13.7 (11.7–17.1)	13.5 (11.5–15.7)	12.9 (11–14.8)	15 (10.2–17.5)	14.6 (12–14.8)
Hematocrit (%)	41.6 (15.9–58)	40.2 (33–48.2)	42.7 (30.7–49.5)	39.3 (32.9–49.4)	39 (31.3–43)	43.1 (32.9–48.7)	43.1 (36–44.9)
Leukocyte count: /mm^3^	3,400 (800–20,100)	5,250 (2,200–9,700)	6,800 (3,400–12,900)	8,100 (5,600–16,500)	5,900 (5,200–8,800)	6,700 (3,300–9,700)	5,000 (2,600–9,300)
Leukopenia*	299 (72)	7 (35)	2 (18)	0 (0)	0 (0)	2 (40)	2 (40)
Leukocytosis*	3 (1)	0 (0)	1 (9)	1 (10)	0 (0)	0 (0)	0 (0)
**Differential count**							
Neutrophil (%)	62 (3–94.1)	71.2 (53–80)	82.6 (59–87.3)	90.1 (64–94)	72 (63–84)	69 (60.3–84.6)	82.7 (73.2–89.3)
Neutrophil count	1,857 (132–15,275)	3,821 (1,306–7,566)	4,573 (2,261–10,965)	6,272 (4,877–15,031)	4,956 (3,276–6,318)	4,040 (2,791–6,693)	3,689 (1,903–6,340)
Granulocytopenia	109 (26)	2 (10)	0 (0)	0(0)	0 (0)	0 (0)	0 (0)
Granulocytosis	42 (10)	1 (5)	6 (55)	6 (60)	2 (29)	1 (20)	3 (60)
Lymphocyte (%)	26.2 (4–77.8)	21.8 (13–41)	13 (6.2–31)	7 (3.2–28.4)	15.4 (10–28)	19 (11.7–31.7)	8.5 (5.3–16.3)
Lymphocyte absolute	885 (192–7,104)	1,261 (504–2,280)	915 (415–1,677)	604 (243–2,783)	1,216 (590–1,456)	1,843 (386–2,123)	400 (327–430)
Lymphocytopenia	105 (25)	4 (20)	8 (73)	7 (70)	3 (43)	1 (20)	4 (80)
Lymphocytosis	91 (22)	2 (10)	0 (0)	0 (0)	0 (0)	0 (0)	0 (0)
Platelet count	88,350 (10,000–452,000)	98,800 (54,000–268,000)	185,000 (158,700–288,000)	101,500 (45,000–185,000)	143,300 (82,000–264,000)	147,000 (67,700–319,000)	105,200 (48,500–151,000)
Platelet <100,000/mm^3^	256 (62)	11 (55)	0 (0)	5 (50)	1 (14)	2 (40)	2 (40)
Platelet <150,000/mm^3^	355 (86)	17 (85)	0 (0)	7 (70)	4 (57)	3 (60)	4 (80)
Thrombocytopenia (150,000) AND leukopenia (<5000)	306 (74)	8 (40)	0 (0)	0 (0)	0 (0)	2 (40)	2 (40)
**Biochemistry profiles**							
Bilirubin >1 mg/dl	6/363 (2)	2/20 (10)	0 (0)	2/10 (20)	3/7 (43)	0 (0)	0 (0)
Direct bilirubin >0.4 mg/dl	21/363 (6)	6/20 (30)	0 (0)	4/10 (40)	3/7 (43)	0 (0)	0 (0)
Indirect bilirubin>0.6 mg/dl	11/363 (3)	1/20 (5)	0 (0)	2/10 (20)	1/7 (14)	0 (0)	0 (0)
SGOT >45 IU	299/363 (82)	6/20 (30)	3/11 (27)	3/10 (30)	5/7 (71)	0 (0)	0 (0)
SGOT >100 IU	136/363 (37)	2/20 (10)	1/11 (9)	1/10 (10)	2/7 (29)	0 (0)	0 (0)
SGPT >35 IU	179/363 (49)	6/20 (30)	4/11 (36)	3/10 (30)	5/7 (71)	0 (0)	0 (0)
SGPT >100 IU	56/363 (15)	3/20 (15)	0 (0)	0 (0)	1/7 (14)	0 (0)	0 (0)
Urea N >40 mg/dl	8/363 (2)	0 (0)	1/9 (11)	4/10 (40)	0 (0)	0 (0)	0 (0)
Creatinine >1.2 mg/dl	39/363 (11)	0 (0)	2/11 (18)	5/10 (50)	0 (0)	0 (0)	0 (0)

Column 2 shows Dengue cases diagnosed at sites and also confirmed by the reference laboratory. Columns 3–8 shows Dengue cases diagnosed at sites, but confirmed due to another pathogen by the reference laboratory.

### Mutation analysis

**Fig 5:**
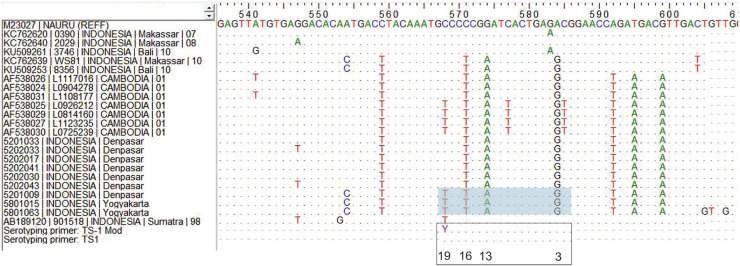
Nucleotide substitutions in TS-1 annealing site (nucleotide 568–586) that affected DENV-1 identification. Alignment of sequences covering the TS1 annealing region of AFIRE DENV-1 with other DENV-1 recently circulating in Indonesia as well as DENV-1 Cambodia (2001), with the prM sequence of DENV-1 Nauru/74 (GenBank Accession No M23027) as numbering reference in positive strand. Dots show similarity of nucleotides in their respective positions. Black arrow shows direction of amplification. Numbers below TS1 Mod sequence indicate nucleotide position where mismatches between the TS-1 primer and viral RNA template occur. The three AFIRE specimens that have the TS-1 mutation are 5201009, 5801015, and 5801063, the TS-1 annealing site is highlighted in grey. The sequences were submitted to GenBank under accession numbers MK634700 to MK634702.

Serotype of dengue could not be determined for three specimens using Dengue serotyping primer set in the study [8], although the specimens produced a valid Dengue group amplicon (511-bp) [8] and Flavivirus group amplicon (266-bp) [17]. Sequencing of the 511-bp amplicon of Dengue group RT-PCR products led to identification of DENV-1 by nucleotide BLAST. Multiple sequence alignment (BioEdit, v7.2.5) to other DENV-1 sequences from GenBank (https://www.ncbi.nlm.nih.gov/genbank/) showed that the three DENV-1 specimens have a C→T mutation at nucleotide 19 of the TS1 primer annealing site (TS1 (568–586) = 5’-CGT CTC AGT GAT CCG GGG G-3’). The mutation prevents amplification by the TS-1 primer required to generate the relevant DENV-1 amplicon (482-bp) (Fig 5). Additional TS1 mutations in our three specimens were identified as TS1_3_(A→G), TS1_13_(G→A), and TS1_16_(C→T).

## Discussion

Our data confirm that dengue is the most common cause of acute febrile illness requiring hospitalization in Indonesia. Our rate of dengue (31.9%) was high relative to previous reports. One study in India did find that DENV infection was responsible for 27% of acute fever syndromes [18]. However, a multicenter surveillance study of acute fever in Asian children showed an incidence of 11.4% [19]. A study in Bandung, Indonesia conducted between 2000–2009 showed 17.4% of febrile cases were due to DENV infection; cases presented with disease severity from DF to DSS [20]. Of note our rate in Bandung was 28% and there was a great deal of variation in rates by region (range 25% - 52%). Our highest rate (52%) occurred in Denpasar, consistent with the Indonesian Ministry of Health dengue data which showed that Bali province had the highest incidence of dengue infection in Indonesia from 2011 to 2015 [21]. Overall rates in our population may differ from previous reports due to inclusion of multiple sites, different settings, diverse age groups, and evolving epidemiology.

Our data also demonstrated that children were exposed to DENV early in life. This finding was consistent across sites. By age 5, more than 40%, and by age of 12, more than 90% showed serologic evidence of prior exposure. Our results are consistent with prior studies of sero-prevalence in Indonesian children, which have shown that 33.8% of children younger than 4 years and 89% of people older than 14 years had been exposed [20, 22]. Though sero-prevalence varies amongst countries [22, 23], other dengue-endemic Asian countries also show high sero-prevalence by adulthood [24–26]. These data are important for vaccine programs as the immunity from previous dengue infection can increase the risk of severe infection. The finding that seronegative vaccinees have an increased risk of hospitalized or severe dengue manifestation starting about 30 months after the first DENV vaccine dose has led the WHO to update its recommendations on DENV vaccination [27–31].

The sensitivity of dengue diagnosis by sites was good, with 88.5% of reference laboratory confirmed dengue cases correctly diagnosed at study sites. On the other hand, 10% of reference laboratory determined non-dengue cases were diagnosed as dengue by study sites. In most non-dengue cases, clinicians diagnosed dengue despite negative dengue rapid tests and/or non-specific clinical presentations. Over-diagnosis of dengue is consistent with prior reports, which showed even higher rates of over-diagnosis during dengue outbreaks [32, 33]. Poor availability of rapid diagnostics for other pathogens may contribute to this phenomenon.

The diagnosis of dengue was missed in 54 (11.5%) cases. In most of these, dengue had been considered and then ruled out based on rapid testing. Clinicians often requested only the NS1 antigen or the IgM/IgG rapid tests. Difficulty in interpreting these results without accurate information about onset of illness may have contributed to the missed diagnoses. In the two fatal cases, comorbidities may have influenced the clinical and laboratory findings, confounding the diagnosis. Most of the missed cases (44/54, 81.5%) were mild (DF), suggesting that in the absence of typical presentations (hemoconcentration, severe thrombocytopenia, bleeding manifestation, or shock), and/or incompletely performed dengue rapid diagnostic test, clinicians may overlook dengue as a cause of fever [34].

Dengue diagnostic inaccuracy may reflect overlap of the dengue clinical syndrome with that of several other infections, suggesting the need for increased awareness of the differential for dengue. Consideration should be given to routine use of diagnostic criteria such as the WHO 2012 criteria [35], which considers clinical parameters, hematology, and dengue diagnostics, to improve diagnostic accuracy. Incorrectly attributing another condition to dengue, which is managed supportively, can result in non-administration of necessary medication and hence worse outcomes. Alternatively, mistaking dengue for bacterial infection can engender unnecessary use of antibiotics and potential development of resistance. Factors contributing to diagnostic inaccuracy merit additional investigation.

DENV infection in Indonesia tended to affect productive age patients and showed a non-significant predisposition for males, consistent with prior reports [21, 36]. A slightly higher proportion of males to females was also observed in the national Indonesia data registry for dengue during each of the 5 data collection years [21].

All four DENV serotypes were identified at all study sites with a predominance of DENV-3 (46.8%) and DENV-1 (26.1%), except in Surabaya where DENV-2 predominated. Previously, Surabaya had shown more DENV-1, which circulated widely in 2012 [37]. Evaluation of monthly DENV serotype distributions at each site demonstrates that the predominant DENV serotype actually is very dynamic (S1 Fig). This may explain differences in predominant DENV serotype in our study compared to previous reports from certain cities. For example, a prior study from Denpasar conducted from March to May 2015, overlapping with our study period, found that DENV-3 is predominant [38]. Our data agree that the predominant serotype during March to May 2015 was DENV-3, though the overall predominant serotype in Denpasar is DENV-1. It has been shown that the predominant serotype may vary between DENV-1 and DENV-3 [20, 37–41], though we found that predominant serotype fluctuates between DENV-1 and DENV-2 in Surabaya. Shifting of predominant serotype is associated with immune alterations in the population [42], although it is unclear if this is occurring in Indonesia. Furthermore, two of our cases showed dual serotype infection, which has been reported in endemic areas [43–45]. This may impact clinical presentation and is considered a major risk factor for dengue with higher severity and mortality [43, 46–48]. Epidemiology and clinical profiles of DENV infection did not vary by serotype in our study, although this has been reported in other studies in Indonesia [4, 37, 38]. High dengue prevalence amongst febrile hospitalized patients, its impact on productive populations and identification of patients with dual dengue infection suggesting that dengue serotypes may circulate simultaneously underscore the need for effective public health approaches to controlling disease.

Severe dengue occurred with secondary DENV-1 and DENV-3 infection. It is known that secondary infections are more likely to be associated with severe disease than primary infections. In a meta-analysis demonstrating that likelihood of severe dengue varies amongst serotypes, primary infection with DENV-3 and secondary infection with DENV-2, DENV-3, and DENV-4 were more likely to result in severe dengue in South East Asia [49] than primary infection with other serotypes or secondary infection with DENV-1. Our results suggest that secondary DENV-1 infection might be more likely than previously realized to cause severe dengue in Indonesia.

Characterization of the DENV E gene informs transmission dynamics, as genotypic variation within serotypes can be a major determinant of epidemics [50]. Shift in genotype predominance has been associated with alterations in immunity against infecting virus and mosquito transmission patterns [51]. During our three-year study (2013–2016), we identified the circulation of both the predominant DENV-1 genotype I and DENV-1 genotype IV. This is consistent with earlier studies showing predominant circulation of DENV-1 genotype I in the recent years, shifting from predominance of DENV-1 genotype IV in the earlier years [20, 41, 52]. This genotype transition pattern might be associated with the faster replication rate of genotype I compared to genotype IV [41]. There was no change for the circulating DENV-2 and DENV-3 genotypes. DENV-2 genotype Cosmopolitan, the only circulating genotype for DENV-2 in Indonesia [20, 41, 53], was observed in our cohort. The Cosmopolitan genotype has a wide geographical distribution, spanning from Asia to Africa [50]. DENV-3 genotype I, which is endemic in Indonesia, Malaysia, the Philippines and the South Pacific Islands [14], and has been shown to circulate dating back to the 1970s, was the only DENV-3 genotype found in this study. Besides DENV-4 genotype IIa that circulated at all sites, a specimen collected from Bali in 2015 was grouped into DENV-4 genotype I, suggesting the first observation of DENV-4 genotype I in Indonesia. DENV-4 genotype I was identified in the Philippines, Thailand, Malaysia, and Srilanka during 1960s-1980s. The observation of DENV-4 genotype I in Bali marks the introduction or possible reemergence of an old strain. This observation merits deeper investigation of the circulation of DENV-4 as genotype shift is closely related to evolving epidemic characteristics.

We herein report DENV-1 virus with TS1_3_(A→G), TS1_13_(G→A), TS1_16_(C→T), and TS1_19_(C→T) substitutions in the annealing site of the TS1 primer (nucleotide 568–586). TS1_19_(C→T) prevents successful DENV-1 serotype determination, as also observed from DENV-1 strains in Cambodia [16]. We indeed observed TS1_19_(C→T) in DENV-1 from a 1998 DHF case in Indonesia (DENV-1 98901518, GenBank accession number AB189120). However, TS1_19_(C→T) that was identified in three DENV-1 from this study (5201009, 5801015, 5801063) are not identical to 98901518 since they have three additional mutations within the TS1 annealing site that were not observed in 98901518. Continuous monitoring for genomic mutations may be warranted because failure in serotype determination might mask the true prevalence of circulating DENV. Clinical implications of TS1_19_(C→T) substitutions were not evident based on our data.

Our study had several limitations. The study was not designed as a dengue evaluation and included only febrile hospitalized patients. Since dengue can be subclinical or mild, and may not require hospitalization, generalizability of our findings is limited. Furthermore, management of cases was determined by the site clinicians; it was not standardized nor was it assessed in detail. Thus, it is difficult to infer causality with regards to patient outcomes. However, our study may be the first to evaluate in detail the challenges of confirming dengue in a resource limited setting with multiple other endemic pathogens that have overlapping manifestations. Finally, as the mutation rate of DENV is high, the gel-based RT_PCR method that we used might miss several dengue cases. This might also be one of the reasons that we could not determine DENV serotypes in 72 subjects besides the viremic phase that had passed when blood was collected. However, the serotype detection rate (85%) in our study was comparable with other studies that used RT-PCR method [54, 55].

DENV is a common etiology of acute fever amongst patients presenting for hospitalization in Indonesia. DENV infection diagnostic accuracy at clinical sites merits optimization since misdiagnosis of DENV infection can adversely impact management and outcomes. Furthermore, mutations may confound diagnosis. Clinicians should consider following diagnostic algorithms that include DENV confirmatory testing and policymakers should prioritize development of laboratory capacity for diagnosis of DENV and other common pathogens.

## Supporting information

S1 Checklist STROBE Checklist. (DOC)

S1 Fig Monthly DENV serotype distribution at each city. Blue: DENV-1. Orange: DENV-2. Grey: DENV-3. Yellow: DENV-4.
(TIF)

S1 Table Table of Oligonucleotide primers used for PCR, and sequencing of DENV 1–4 structural genes (C,prM/M, E). (DOCX)

S2 Table Clinical symptoms at enrolment by DENV serotypes. (DOCX)

S1 Dataset (XLSX)

## References

[1] WHO. Global Strategy for Dengue Prevention and Control 2012–2020. World Health Organization [Internet] 2012 [cited 2017 Sep 28];43 Available from: www.who.int/neglected_diseases/en.

[2] Bhatt S, Gething PW, Brady OJ, Messina JP, Farlow AW, Moyes CL, et al The global distribution and burden of dengue. Nature. 2013;496(7446):504–7. 10.1038/nature12060 23563266 PMC3651993

[3] Hales S, de Wet N, Maindonald J, Woodward A. Potential effect of population and climate changes on global distribution of dengue fever: an empirical model. Lancet. 2002;360(9336):830–4. 10.1016/S0140-6736(02)09964-6 . 12243917

[4] Karyanti MR, Uiterwaal CS, Kusriastuti R, Hadinegoro SR, Rovers MM, Heesterbeek H, et al The changing incidence of dengue haemorrhagic fever in Indonesia: a 45-year registry-based analysis. BMC Infect Dis. 2014;14(1):412. 25064368 10.1186/1471-2334-14-412 PMC4122763

[5] WHO SEARO. Comprehensive Guidelines for Prevention and Control of Dengue and Dengue Haemorrhagic Fever: WHO SEARO; 2011.

[6] Karyana M, Kosasih H, Samaan G, Tjitra E, Aman AT, Alisjahbana B, et al INA-RESPOND: a multi-centre clinical research network in Indonesia. Health Res Policy Syst. 2015;13:34 10.1186/s12961-015-0024-9 26219280 PMC4518592

[7] Focusdx.com. Dengue Virus Serology Testing. Focusdxcom 2016 [cited 2017 Sep 28] Available from: https://www.focusdx.com/pdfs/brochures/DXDENI0511_Dengue_Virus_Serology.pdf.

[8] Lanciotti RS, Calisher CH, Gubler DJ, Chang G-J, Vorndam AV. Rapid detection and typing of dengue viruses from clinical samples by using reverse transcriptase-polymerase chain reaction. J Clin Microbiol. 1992;30(3):545–51. 1372617 10.1128/jcm.30.3.545-551.1992 PMC265106

[9] Zhang C, Mammen MP, Chinnawirotpisan P, Klungthong C, Rodpradit P, Monkongdee P, et al Clade replacements in dengue virus serotypes 1 and 3 are associated with changing serotype prevalence. J Virol. 2005;79(24):15123–30. 10.1128/JVI.79.24.15123-15130.2005 16306584 PMC1316048

[10] Zhang C, Mammen MP Jr., Chinnawirotpisan P, Klungthong C, Rodpradit P, Nisalak A, et al Structure and age of genetic diversity of dengue virus type 2 in Thailand. J Gen Virol. 2006;87(Pt 4):873–83. 10.1099/vir.0.81486-0 . 16528037

[11] Goncalvez AP, Escalante AA, Pujol FH, Ludert JE, Tovar D, Salas RA, et al Diversity and evolution of the envelope gene of dengue virus type 1. Virology. 2002;303(1):110–9. 10.1006/viro.2002.1686 12482662

[12] Christenbury JG, Aw PP, Ong SH, Schreiber MJ, Chow A, Gubler DJ, et al A method for full genome sequencing of all four serotypes of the dengue virus. J Virol Methods. 2010;169(1):202–6. 10.1016/j.jviromet.2010.06.013 . 20600330

[13] Twiddy SS, Farrar JJ, Chau NV, Wills B, Gould EA, Gritsun T, et al Phylogenetic relationships and differential selection pressures among genotypes of dengue-2 virus. Virology. 2002;298(1):63–72. 10.1006/viro.2002.1447 12093174

[14] Lanciotti RS, Lewis JG, Gubler DJ, Trent DW. Molecular evolution and epidemiology of dengue-3 viruses. J Gen Virol. 1994;75(1):65–75. 8113741 10.1099/0022-1317-75-1-65

[15] AbuBakar S, Wong PF, Chan YF. Emergence of dengue virus type 4 genotype IIA in Malaysia. J Gen Virol. 2002;83(Pt 10):2437–42. 10.1099/0022-1317-83-10-2437 . 12237425

[16] Reynes JM, Ong S, Mey C, Ngan C, Hoyer S, Sall AA. Improved molecular detection of dengue virus serotype 1 variants. J Clin Microbiol. 2003;41(8):3864–7. 10.1128/JCM.41.8.3864-3867.2003 12904404 PMC179838

[17] Kuno G, Chang G-JJ, Tsuchiya KR, Karabatsos N, Cropp CB. Phylogeny of the genus Flavivirus. J Virol. 1998;72(1):73–83. 9420202 10.1128/jvi.72.1.73-83.1998 PMC109351

[18] Rani R, Sundararajan T, Rajesh S, Jeyamurugan T. A study on common etiologies of acute febrile illness detectable by microbiological tests in a tertiary care hospital. Int J Curr Microbiol App Sci. 2016;5(7):670–4.

[19] Capeding MR, Chua MN, Hadinegoro SR, Hussain II, Nallusamy R, Pitisuttithum P, et al Dengue and other common causes of acute febrile illness in Asia: an active surveillance study in children. PLoS Negl Trop Dis. 2013;7(7):e2331 10.1371/journal.pntd.0002331 23936565 PMC3723539

[20] Kosasih H, Alisjahbana B, de Mast Q, Rudiman IF, Widjaja S, Antonjaya U, et al The epidemiology, virology and clinical findings of dengue virus infections in a cohort of Indonesian adults in Western Java. PLoS Negl Trop Dis. 2016;10(2):e0004390 10.1371/journal.pntd.0004390 26872216 PMC4752237

[21] Kementerian Kesehatan Indonesia. Situasi DBD di Indonesia. Info Datin [Internet] 2016 [cited 2017 Sep 26]; Available from: http://www.depkes.go.id/download.php?file=download/pusdatin/infodatin/infodatin-demam-berdarah.pdf

[22] Alera MT, Srikiatkhachorn A, Velasco JM, Tac-An IA, Lago CB, Clapham HE, et al Incidence of dengue virus infection in adults and children in a prospective longitudinal cohort in the Philippines. PLoS Negl Trop Dis. 2016;10(2):e0004337 10.1371/journal.pntd.0004337 26845762 PMC4742283

[23] Ang LW, Cutter J, James L, Goh KT. Seroprevalence of past dengue virus infection among children and adolescents in Singapore. J Med Virol. 2015;87(12):2159–62. 10.1002/jmv.24287 26058712

[24] Chew CH, Woon YL, Amin F, Adnan TH, Wahab AHA, Ahmad ZE, et al Rural-urban comparisons of dengue seroprevalence in Malaysia. BMC Public Health. 2016;16(1):824 10.1186/s12889-016-3496-9 27538986 PMC4991088

[25] Mohsin S, Ghafoor F, Saleem M, Ghous R, Aasim M. Seroprevalence of asymptomatic dengue infection in children in Lahore. Epidemiol Infect. 2016;144(11):2276–82. 10.1017/S0950268816000522 27019361 PMC9150536

[26] Thai KT, Binh TQ, Giao PT, Phuong HL, Hung LQ, Nam NV, et al Seroprevalence of dengue antibodies, annual incidence and risk factors among children in southern Vietnam. Trop Med Int Health. 2005;10(4):379–86. 10.1111/j.1365-3156.2005.01388.x 15807802

[27] WHO. Revised SAGE recommendation dengue vaccine 2018 [cited 2018 28 April]. Available from: http://www.who.int/immunization/diseases/dengue/revised_SAGE_recommendation_dengue_vaccine_apr2018/en/. 10.1016/j.vaccine.2018.09.063

[28] Normile D. Safety concerns derail dengue vaccination program. Science. 2017;358(6370):1514–5. 10.1126/science.358.6370.1514 . 29269451

[29] Dyer O. Philippines halts dengue immunisation campaign owing to safety risk. BMJ. 2017;359:j5759 10.1136/bmj.j5759 . 29233814

[30] Katzelnick LC, Gresh L, Halloran ME, Mercado JC, Kuan G, Gordon A, et al Antibody-dependent enhancement of severe dengue disease in humans. Science. 2017;358(6365):929–32. 10.1126/science.aan6836 29097492 PMC5858873

[31] Hadinegoro SR, Arredondo-Garcia JL, Capeding MR, Deseda C, Chotpitayasunondh T, Dietze R, et al Efficacy and Long-Term Safety of a Dengue Vaccine in Regions of Endemic Disease. N Engl J Med. 2015;373(13):1195–206. 10.1056/NEJMoa1506223 . 26214039

[32] Suwandono A, Kosasih H, Nurhayati, Kusriastuti R, Harun S, Ma'roef C, et al Four dengue virus serotypes found circulating during an outbreak of dengue fever and dengue haemorrhagic fever in Jakarta, Indonesia, during 2004. Trans R Soc Trop Med Hyg. 2006;100(9):855–62. 10.1016/j.trstmh.2005.11.010 . 16507313

[33] Suharti C, van Gorp EC, Dolmans WM, Groen J, Hadisaputro S, Djokomoeljanto RJ, et al Hanta virus infection during dengue virus infection outbreak in Indonesia. Acta Med Indones. 2009;41(2):75–80. . 19390126

[34] Centers for Disease Control and Prevention. Underdiagnosis of dengue—Laredo, Texas, 1999. JAMA. 2001;285(7):877 . 11236798

[35] WHO. Handbook for clinical management of dengue. Geneva: WHO 2012 [cited 2017 Sep 26] 114 p Available from: http://www.whoint/about/licensing/copyright_form/en/indexhtml. 2012.

[36] Anker M, Arima Y. Male–female differences in the number of reported incident dengue fever cases in six Asian countries. Western Pacific Surveillance and Response Journal: WPSAR. 2011;2(2):17 10.5365/WPSAR.2011.2.1.002 23908884 PMC3730962

[37] Wardhani P, Aryati A, Yohan B, Trimarsanto H, Setianingsih TY, Puspitasari D, et al Clinical and virological characteristics of dengue in Surabaya, Indonesia. PLoS One. 2017;12(6):e0178443 10.1371/journal.pone.0178443 28575000 PMC5456069

[38] Megawati D, Masyeni S, Yohan B, Lestarini A, Hayati RF, Meutiawati F, et al Dengue in Bali: Clinical characteristics and genetic diversity of circulating dengue viruses. PLoS Negl Trop Dis. 2017;11(5):e0005483 10.1371/journal.pntd.0005483 28531223 PMC5456401

[39] Fahri S, Yohan B, Trimarsanto H, Sayono S, Hadisaputro S, Dharmana E, et al Molecular surveillance of dengue in Semarang, Indonesia revealed the circulation of an old genotype of dengue virus serotype-1. PLoS Negl Trop Dis. 2013;7(8):e2354 10.1371/journal.pntd.0002354 23951374 PMC3738473

[40] Graham R, Juffrie M, Tan R, Hayes C, Laksono I, Ma'Roef C, et al A prospective seroepidemiologic study on dengue in children four to nine years of age in Yogyakarta, Indonesia I. studies in 1995–1996. Am J Trop Med Hygiene. 1999;61(3):412–9. 10.4269/ajtmh.1999.61.412 10497982

[41] Sasmono RT, Wahid I, Trimarsanto H, Yohan B, Wahyuni S, Hertanto M, et al Genomic analysis and growth characteristic of dengue viruses from Makassar, Indonesia. Infect Genet Evol. 2015;32:165–77. 10.1016/j.meegid.2015.03.006 25784569

[42] Adams B, Holmes EC, Zhang C, Mammen MP Jr., Nimmannitya S, Kalayanarooj S, et al Cross-protective immunity can account for the alternating epidemic pattern of dengue virus serotypes circulating in Bangkok. Proc Natl Acad Sci U S A. 2006;103(38):14234–9. 10.1073/pnas.0602768103 16966609 PMC1599940

[43] Bharaj P, Chahar HS, Pandey A, Diddi K, Dar L, Guleria R, et al Concurrent infections by all four dengue virus serotypes during an outbreak of dengue in 2006 in Delhi, India. Virology. 2008;5(1):1. 10.1186/1743-422X-5-1 PMC2253528 18182120

[44] Lardo S, Utami Y, Yohan B, Tarigan SM, Santoso WD, Nainggolan L, et al Concurrent infections of dengue viruses serotype 2 and 3 in patient with severe dengue from Jakarta, Indonesia. Asian Pac J Trop Med. 2016;9(2):134–40. 10.1016/j.apjtm.2016.01.013 26919942

[45] Lorono-Pino M, Cropp C, Farfan J, Vorndam A, Rodriguez-Angulo E, Rosado-Paredes E, et al Common occurrence of concurrent infections by multiple dengue virus serotypes. Am J Trop Med Hyg. 1999;61(5):725–30. 10.4269/ajtmh.1999.61.725 10586902

[46] Dhanoa A, Hassan SS, Ngim CF, Lau CF, Chan TS, Adnan NAA, et al Impact of dengue virus (DENV) co-infection on clinical manifestations, disease severity and laboratory parameters. BMC Infect Dis. 2016;16(1):406 10.1186/s12879-016-1731-8 27514512 PMC4982428

[47] Kuno G. Emergence of the severe syndrome and mortality associated with dengue and dengue-like illness: historical records (1890 to 1950) and their compatibility with current hypotheses on the shift of disease manifestation. Clin Microbiol Rev. 2009;22(2):186–201. 10.1128/CMR.00052-08 19366911 PMC2668235

[48] Vinodkumar C, Kalapannavar N, Basavarajappa K, Sanjay D, Gowli C, Nadig NG, et al Episode of coexisting infections with multiple dengue virus serotypes in central Karnataka, India. J Infect Public Health. 2013;6(4):302–6. 10.1016/j.jiph.2013.01.004 23806706

[49] Soo K-M, Khalid B, Ching S-M, Chee H-Y. Meta-analysis of dengue severity during infection by different dengue virus serotypes in primary and secondary infections. PLoS One. 2016;11(5):e0154760 10.1371/journal.pone.0154760 27213782 PMC4877104

[50] Wei K, Li Y. Global evolutionary history and spatio-temporal dynamics of dengue virus type 2. Sci Rep. 2017;7:45505 10.1038/srep45505 28378782 PMC5381229

[51] Lambrechts L, Fansiri T, Pongsiri A, Thaisomboonsuk B, Klungthong C, Richardson JH, et al Dengue-1 virus clade replacement in Thailand associated with enhanced mosquito transmission. J Virol. 2012;86(3):1853–61. 10.1128/JVI.06458-11 22130539 PMC3264336

[52] Kotaki T, Yamanaka A, Mulyatno KC, Churrotin S, Labiqah A, Sucipto TH, et al Continuous dengue type 1 virus genotype shifts followed by co-circulation, clade shifts and subsequent disappearance in Surabaya, Indonesia, 2008–2013. Infect Genet Evol. 2014;28:48–54. 10.1016/j.meegid.2014.09.002 25219342

[53] Kotaki T, Yamanaka A, Mulyatno KC, Churrotin S, Sucipto TH, Labiqah A, et al Divergence of the dengue virus type 2 Cosmopolitan genotype associated with two predominant serotype shifts between 1 and 2 in Surabaya, Indonesia, 2008–2014. Infect Genet Evol. 2016;37:88–93. 10.1016/j.meegid.2015.11.002 26553170

[54] Yousseu FBS, Nemg FBS, Ngouanet SA, Mekanda FMO, Demanou M. Detection and serotyping of dengue viruses in febrile patients consulting at the New-Bell District Hospital in Douala, Cameroon. PLoS One. 2018;13(10):e0204143 10.1371/journal.pone.0204143 30281633 PMC6169880

[55] Kusmintarsih ES, Hayati RF, Turnip ON, Yohan B, Suryaningsih S, Pratiknyo H, et al Molecular characterization of dengue viruses isolated from patients in Central Java, Indonesia. J Infect Public Health. 2018;11(5):617–25. 10.1016/j.jiph.2017.09.019 . 29056517

