# Supplementary figures and images for: Dengue viral infection in Indonesia: Epidemiology, diagnostic challenges, and mutations from an observational cohort study
Source: PLoS Negl Trop Dis. 2019 Oct 21;13(10):e0007785. doi: 10.1371/journal.pntd.0007785 (PMC6822776; doi:10.1371/journal.pntd.0007785)

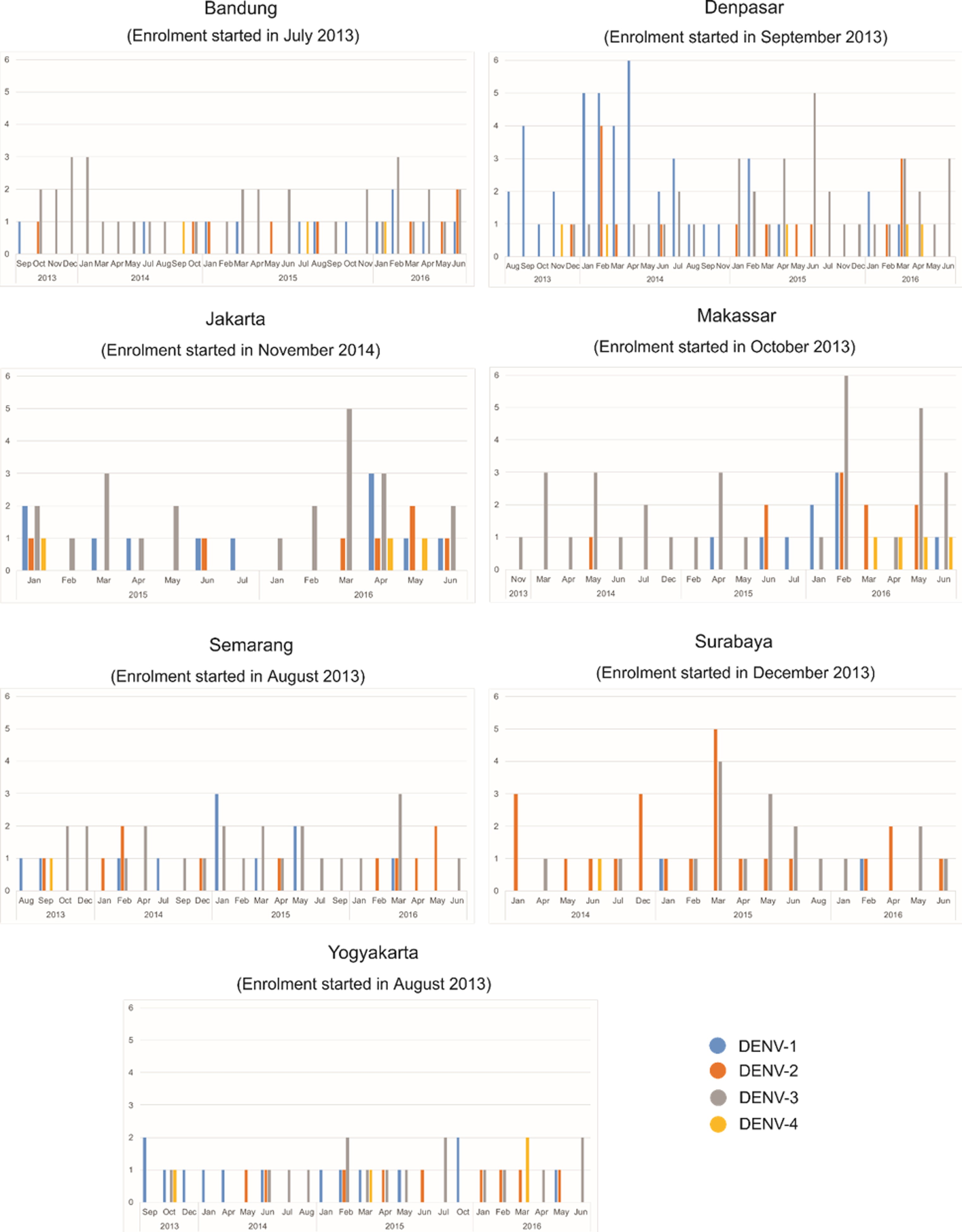

Supplement: S1 Fig — Blue: DENV-1. Orange: DENV-2. Grey: DENV-3. Yellow: DENV-4. (TIF) [file pntd.0007785.s002.tif]
