# Supplementary material for: Dengue viral infection in Indonesia: Epidemiology, diagnostic challenges, and mutations from an observational cohort study
Source: PLoS Negl Trop Dis. 2019 Oct 21;13(10):e0007785. doi: 10.1371/journal.pntd.0007785 (PMC6822776; doi:10.1371/journal.pntd.0007785)
Supplement: S1 Table — (DOCX) [file pntd.0007785.s003.docx]

**Supplementary Table 1. Table of oligonucleotide primers used for PCR, and sequencing of DENV 1-4 structural genes (C, prM/M, E).**

| Serotype | Primer name | Sequence (5' - 3') | Annealing position |
| --- | --- | --- | --- |
| DENV-1 | D1F751 | GGATGTCCTCTGAAGGCGC | 751-769 |
| DENV-1 | D1F1035 | CCAACATTGGACATTGAAC | 1035-1053 |
| DENV-1 | D1F1545 | CACAAACAATGGTTTCTAG | 1545-1563 |
| DENV-1 | D1R1653 | CATGAGCTGTCTT(A,G)AATGTG | 1634-1653 |
| DENV-1 | D1R2297 | CCCTATTCCTATTTTCATGG | 2278-2297 |
| DENV-1 | D1R2581 | GCTGATCGAATTCCACACAC | 2562-2581 |
| DENV-2 | D2F798 | CCAGAGAATTGAAACTTGGA | 798-817 |
| DENV-2 | D2F1200 | GAGGTTCGTCTGCAAACA | 1200-1217 |
| DENV-2 | D2F1729 | ACAGGAGCCACAGAAATCCA | 1729-1748 |
| DENV-2 | D2R1667 | AGGGGATTTTTGAAAGTG | 1650-1667 |
| DENV-2 | D2R2099 | TTCAGTTGTCCCGGCTCT | 2080-2099 |
| DENV-2 | D2R2516 | GTATTGTTCTGTCCATGTGT | 2495-2516 |
| DENV-3 | D3F791 | CAAGTCGAGAAGGTAGAGAC | 791-810 |
| DENV-3 | D3F1187 | GAGCAGGACCAGAACTACGT | 1187-1206 |
| DENV-3 | D3F1667 | AAGCAAGAAGTAGTTGTCCT | 1667-1686 |
| DENV-3 | D3F1927 | CAAGATTCCTTTCTCCAC | 1927-1944 |
| DENV-3 | D3R1340 | TGAGGTTCTCATGTTGCACC | 1323-1340 |
| DENV-3 | D3R1758 | CTTGTGCCTCCTGAGGTTTG | 1739-1758 |
| DENV-3 | D3R2095 | GATTTTCAGGGCTTTGTC | 2078-2095 |
| DENV-3 | D3R2492 | TGTGGACCTCATTAGTGACG | 2473-2492 |
| DENV-4 | d4s1c | AGTTGTTAGTCTGTGTGGACCGAC | 1-24 |
| DENV-4 | d4s2 | AACAAATGCACYCTYATTGCCA | 534-555 |
| DENV-4 | d4s3 | TTTGAACTGACYAAGACAACAGC | 1065-1087 |
| DENV-4 | d4s4 | TGGACAGCAGGAGCAGACACAT | 1596-1617 |
| DENV-4 | d4a6 | TGGGTGTCTCCATTGTGGACTG | 1363-1385 |
| DENV-4 | d4a18 | GGGCATTYAATATTGCAGACGCTA | 2548-2571 |
