# Supplementary material for: Dengue viral infection in Indonesia: Epidemiology, diagnostic challenges, and mutations from an observational cohort study
Source: PLoS Negl Trop Dis. 2019 Oct 21;13(10):e0007785. doi: 10.1371/journal.pntd.0007785 (PMC6822776; doi:10.1371/journal.pntd.0007785)
Supplement: S2 Table — (DOCX) [file pntd.0007785.s004.docx]

**Supplementary Table 2. Clinical symptoms at enrolment by DENV serotypes.**

|  | **DENV-1 (N=103)** | **DENV-2 (N=84)** | **DENV-3 (N=186)** | **DENV-4 (N=21)** |
| --- | --- | --- | --- | --- |
| **General symptoms** |  |  |  |  |
| Anorexia | 37 (35.9) | 28 (33.3) | 55 (29.6) | 8 (38.1) |
| Chills | 12 (11.7) | 16 (19) | 25 (13.4) | 3 (14.3) |
| Pruritus | 0 | 0 | 3 (1.6) | 0 |
| Lethargy | 21 (20.4) | 12 (14.3) | 40 (21.5) | 7 (33.3) |
| Paleness | 1 (1) | 0 | 0 | 1 (4.8) |
| Icterus | 0 | 1 (1.2) | 0 | 0 |
| **Neurological symptoms** |  |  |  |  |
| Headache | 51 (49.5) | 51 (60.7) | 110 (59.1) | 9 (42.9) |
| Dizziness | 22 (21.4) | 14 (16.7) | 38 (20.4) | 4 (19) |
| **Respiratory symptoms** |  |  |  |  |
| Cough | 18 (17.5) | 12 (14.3) | 29 (15.6) | 2 (9.5) |
| Sore Throat | 7 (6.8) | 9 (10.7) | 12 (6.5) | 1 (4.8) |
| Runny Nose | 7 (6.8) | 6 (7.1) | 12 (6.5) | 0 |
| Epistaxis | 7 (6.8) | 7 (8.3) | 9 (4.8) | 2 (9.5) |
| **GI symptoms** |  |  |  |  |
| Constipation | 4 (3.9) | 5 (6) | 7 (3.8) | 0 |
| Abdominal Pain | 20 (19.4) | 11 (13.1) | 29 (15.6) | 3 (14.3) |
| Diarrhea | 10 (9.7) | 7 (8.3) | 16 (8.6) | 3 (14.3) |
| Nausea | 72 (69.9) | 68 (81) | 138 (74.2) | 19 (90.5) |
| Vomiting | 54 (52.4) | 43 (51.2) | 100 (53.8) | 13 (61.9) |
| Epigastric Pain | 24 (23.3) | 15 (17.9) | 50 (26.9) | 1 (4.8) |
| **Urinary symptoms** |  |  |  |  |
| Suprapubic pain | 1 (1) | 1 (1.2) | 0 | 0 |
| Dysuria | 4 (3.9) | 2 (2.4) | 2 (1.1) | 0 |
| **Musculoskeletal symptoms** |  |  |  |  |
| Arthralgia | 34 (33) | 29 (34.5) | 54 (29) | 6 (28.6) |
| Myalgia | 25 (24.3) | 17 (20.2) | 50 (26.9) | 5 (23.8) |
| Ecchymosis | 14 (13.6) | 16 (19) | 28 (15.1) | 1 (4.8) |
